# Potent Anti-Inflammatory Effects of Tetracyclines on Human Eosinophils

**DOI:** 10.3389/falgy.2021.754501

**Published:** 2021-10-04

**Authors:** Manuela Gehring, Dorothea Wieczorek, Alexander Kapp, Bettina Wedi

**Affiliations:** Department of Dermatology and Allergy, Comprehensive Allergy Center, Hannover Medical School, Hanover, Germany

**Keywords:** eosinophils, apoptosis, *Staphylococcus enterotoxin*, allergic inflammation, tetracycline

## Abstract

Eosinophils are potent pro-inflammatory cells. Not only in allergic diseases but also in other diseases there is a need for treatment strategies to induce resolution of eosinophil-mediated inflammation. During the last years beneficial non-antibiotic activities of tetracyclines (TCNs) have been shown in different diseases in which eosinophils play a role, for example, asthma and bullous pemphigoid. The working mechanism of these effects remains to be clarified. Aim of the present study was to investigate the effects of TCNs on eosinophils. Flow cytometry analysis of apoptosis, mitochondrial membrane potential, activation of caspases, intracellular H_2_O_2_ and calcium, surface expression of eosinophil activation markers was performed in highly purified peripheral blood eosinophils of non-atopic donors. Tetracycline hydrochloride, minocycline and doxycycline significantly induced eosinophil apoptosis. All TCNs were able to significantly overcome the strong survival enhancing effects of pro-eosinophilic cytokines and staphylococcus aureus enterotoxins. Tetracycline hydrochloride induced eosinophil apoptosis was accompanied by intracellular production of hydrogen peroxide, loss of mitochondrial membrane potential and activation of caspases. Moreover, tetracycline hydrochloride significantly down regulated eosinophil surface expression of CD9 and CD45, and of the activation markers CD11b and CD69, but not of CD54, CD63, or CD95. Our data, propably for the first time, point to a potent anti-inflammatory role of TCNs on eosinophils.

## Introduction

Eosinophils are multifunctional leukocytes and by modulating innate and adaptive immunity, they play a pro-inflammatory key role in several diseases (1) including parasitic infections (2) allergic diseases such as atopic dermatitis and inhalant allergy (3–7), but also autoimmune diseases such as bullous pemphigoid (8), esophageal (9), and gastrointestinal disorders (10), mental disorders (11) and cancer (12–14). The downregulation of pro-inflammatory activities and induction of apoptosis in eosinophils promotes the resolution of inflammation. Until now, only few promoters of eosinophil apoptosis aside from glucocorticosteroids (15, 16) have been described, for example, theophylline (17) transforming growth factor-ß (18, 19), abrogation of Fas/CD95 by its ligand or a monoclonal antibody (mAb) (20, 21) or CD69 perturbation with mAb (22). Unfortunately, most of these are not available as prescription drugs or have potent adverse effects.

Tetracyclines (TCNs) were discovered in the 1940s and are broad-spectrum antibiotics that act as such at the ribosomal level where they interfere with protein synthesis (23). Dermatologists are using TCNs since the 1950s to treat disorders that do not necessarily have an infectious etiology (24). In recent years, several studies have conclusively reported non-antibiotic activities of TCNs including anti-inflammatory, immune-modulating and neuroprotective properties and many clinical trials are ongoing over a wide range of diseases including dermatological diseases, behavior and mental disorders, immune system disorders, cardiovascular diseases, and cancer (25). For example, the BLISTER study has shown that starting patients on doxycycline is non-inferior to standard treatment with oral prednisolone for short-term blister control in bullous pemphigoid and significantly safer in the long-term (26). A recent systematic review and network meta-analysis concluded that combined doxycycline and nicotinamides are a safer and more effective option for extensive bullous pemphigoid patients as compared to usual use of systemic steroids and that minocycline may be a safer promising option for renal impairment patients (27). The beneficial non-antibiotic properties of TCNs in bullous pemphigoid and other diseases are far from being clear. The prominent deleterious role of eosinophils in bullous pemphigoid is well-established (28–31).

Therefore, our aim was to investigate whether TCNs modulate eosinophil function and survival.

## Materials and Methods

### Reagents

If not otherwise stated, reagents were obtained from Sigma-Aldrich Chemicals, Schnelldorf, Germany. Pro-eosinophilic cytokine mix consisted of Interleukin (IL)-3, IL-5, and GM-CSF, 10 ng/ml each (R&D Systems, Wiesbaden, Germany). *Staphylococcus aureus* enterotoxins (SE) of maximal purity (without LPS contamination), SEA, SEB, and SEC were obtained from Toxin Technology, Inc (Sarasota, Fla).

### Purification of Eosinophils

Peripheral blood eosinophils from healthy non-atopic volunteers after informed consent (approved by the ethics committee of the Hannover Medical School) were either separated by Ficoll density gradient centrifugation and an improved immunomagnetic negative selection procedure using anti CD16 antibody-coated Dynabeads (Dynal A.S., Oslo, Norway) as described in detail (32–34) or were isolated using a magnetic cell separation system (MACS) according to the instructions of the manufacturer (Milteny Biotec, Bergisch Gladbach, Germany). Purity and viability were 96% or greater after isolation, as assessed by Kimura staining and trypan blue dye exclusion, respectively.

### Modified Nicoletti's Protocol and Mitochondrial Membrane Potential

The proportion of eosinophils displaying a hypodiploid DNA peak was determined using a modification of the protocol of Nicoletti as described (35–37). In brief, 1 × 10^5^ eosinophils were resuspended in 200 μl of hypotonic fluorochrome solution (propidium iodide, 50 μg/ml in 0.1% sodium citrate plus 0.1% Triton X-100) and incubated for 2 h at 4°C. Apoptotic eosinophil nuclei were distinguished by their hypodiploid DNA content from the diploid DNA content of normal eosinophil nuclei. For inhibition of apoptosis eosinophils were pre-incubated for 2 h at 37°C with 50 μM of pan-caspase inhibitor (zVAD-fmk; R&D Systems, MN, USA) and then washed and stimulated with tetracycline hydrochloride (5 × 10^−4^ M and 10^−4^ M), and dexamethasone (10^−6^ M) for 24 h. For mitochondrial membrane potential detection 1 × 10^6^ eosinophils were incubated with 10 mM JC-1 (Molecular Probes, Eugene, OR) at room temperature for 20 min, centrifuged, washed twice with 10 x assay buffer, and analyzed by flow cytometry. In healthy cells with high mitochondrial membrane potential (ΔΨm), JC-1 spontaneously forms complexes known as J-aggregates with intense red fluorescence 2 (~590 nm). In apoptotic cells with low ΔΨm, JC-1 remains in the monomeric form, which shows only green fluorescence 1 (~525 nm). The ratio of green to red fluorescence is dependent only on the membrane potential.

### Flow Cytometric Analysis of Intracellular H_2_O_2_, Calcium Mobilization and Surface Antigens

To measure changes in intracellular H_2_O_2_, we used the oxidation-sensitive fluorescent probe dihydrorhodamine (DHR; Molecular Probes, Eugene, Oregon, U.S.A.). Eosinophils (1 x 10^6^/ml) were incubated with control medium, dexamethasone (10^−6^ M) or tetracycline hydrochloride as indicated for 2 and 24 h at 37°C. Thereafter the cells were labeled with 1 μM DHR for another 10 min at 37°C. PMA (10^−7^ M) was used as positive control. Fluo-4 AM is essentially non-fluorescent in the absence of Ca2+ and exhibits an increase in fluorescence emission upon binding Ca2+. 1 × 106 eosinophils/ml in calcium influx buffer (PBS supplemented with 1 mM calcium chloride, 1 mM magnesium chloride, and 1% bovine serum albumin) were loaded with 5 μM Fluo-4 AM (Molecular Probes, Karlsruhe, Germany) for 30 min at 37°C in the dark. After washing and centrifugation measurement was performed in the FLUOstar plate reader (BMG Lab Technologies, Offenburg, Germany). After 5 s stimulation with tetracycline hydrochloride (5 × 10^−4^ M), C5a (10^−8^ M, positive control), or medium were done and changes in cellular fluorescence (excitation, 485 nm; emission, 520 nm) was recorded per second for 50 s. To characterize the effect of tetracycline hydrochloride on surface antigen expression single color immunofluorescence technique was performed as described previously (33). Surface antigen staining was performed after stimulation of eosinophils with control medium, IL-3 (10 ng/ml), dexamethasone (10^−6^ M) or tetracycline hydrochloride (10^−3^ M, 5 × 10^−4^ M, 10^−4^ M) for 24 and 48 h.

### Statistics

Unless otherwise stated all data are presented as mean ± SEM. To see if the effect of stimulation was significant, paired *t-*test or Wilcoxon signed Rank Test were used, depending on the distribution of the data, a statistical software package (SigmaStat for Windows, Jandel Scientific, Erkrath, Germany) being used. A *P*-value < 0.05 was considered statistically significant.

## Results

### TCNs Induced Caspase-Dependent Eosinophil Apoptosis Preceded by Mitochondrial Damage

**Figure 1:**
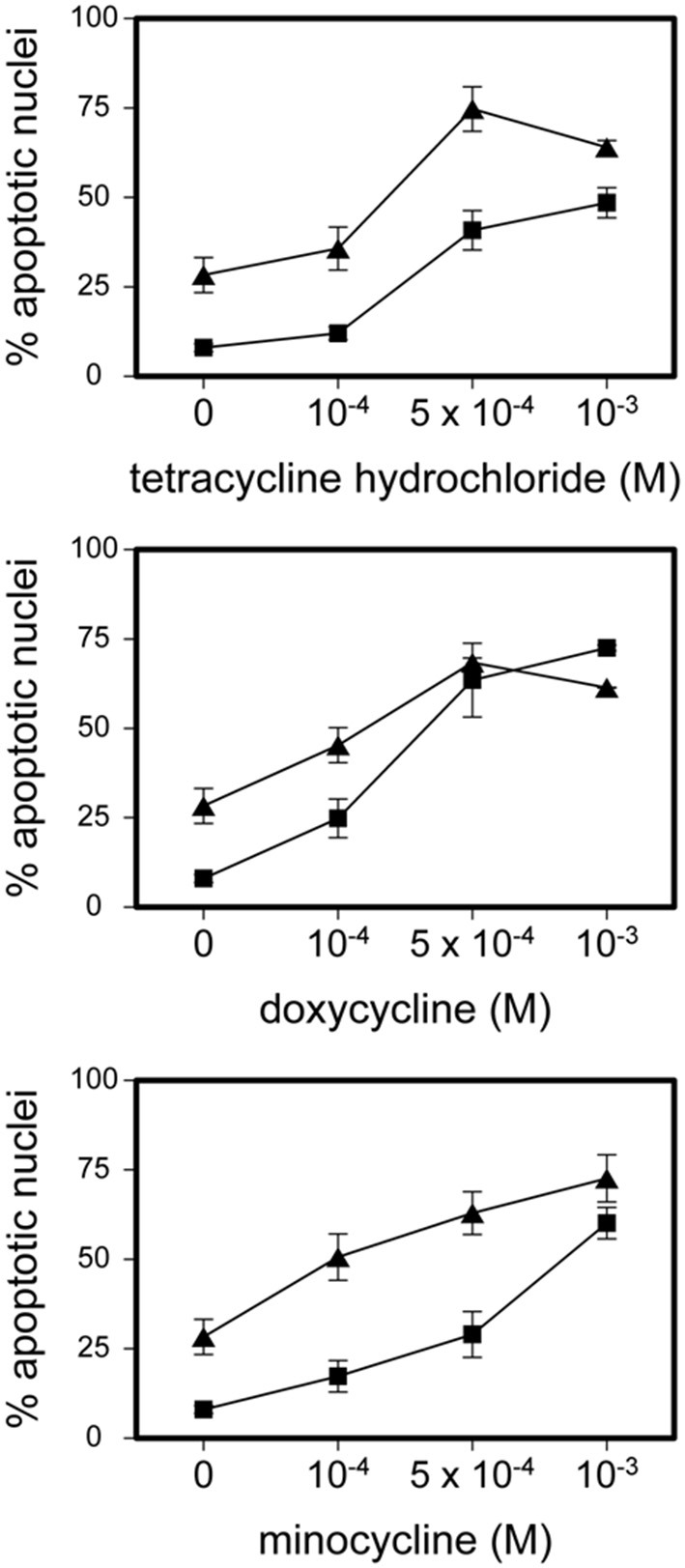
Tetracycline hydrochloride, minocycline and doxycycline induced eosinophil apoptosis. Stimulation of eosinophils with indicated TCNs at 10^−4^ M up to 10^−3^ M for 24 h (■) and 48 h (▲) (*p* < 0.05 up to *p* < 0.001 vs. medium control = 0). Mean percentage of apoptotic nuclei ± SEM (*n* = 9). Representative histogram analysis.

**Figure 2:**
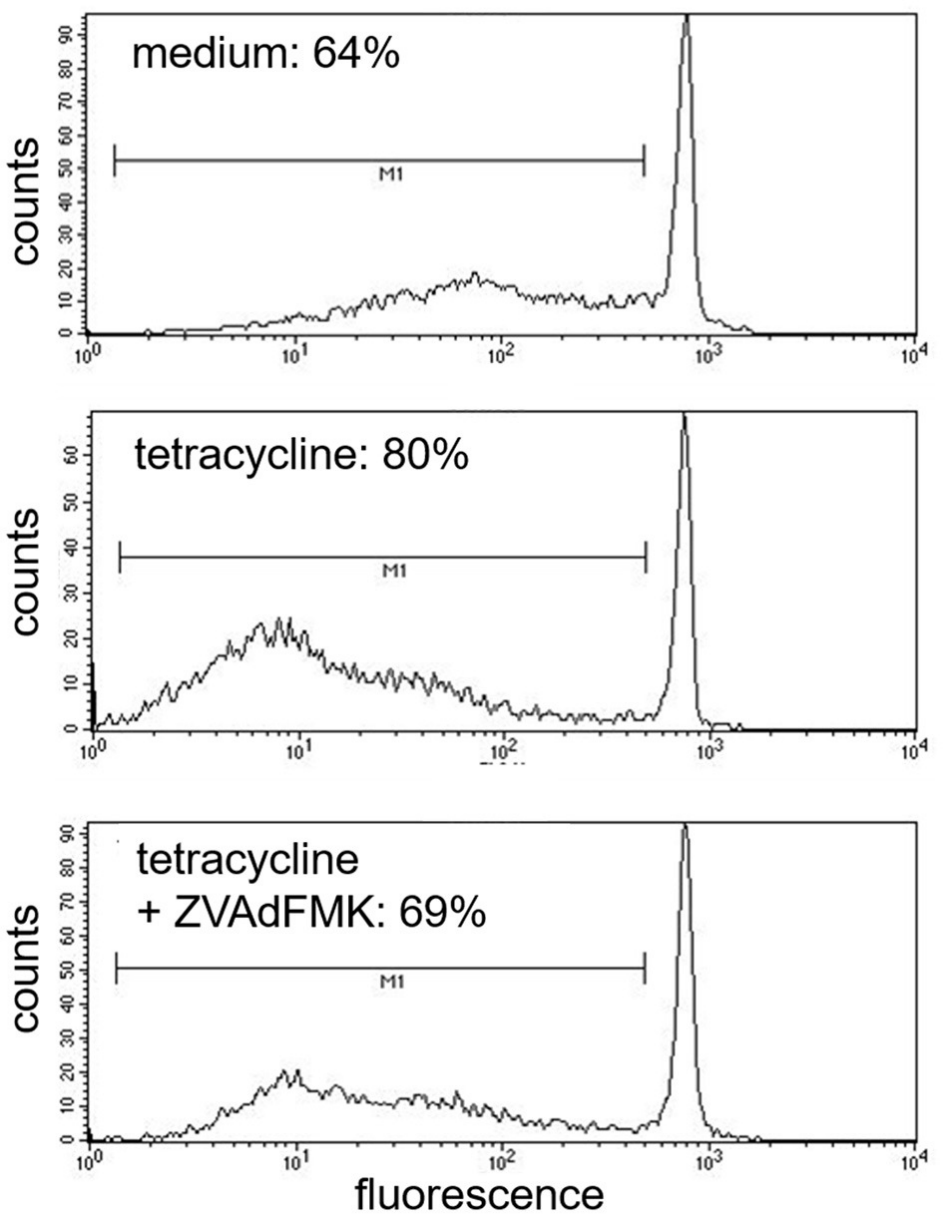
Inhibition of tetracycline-induced apoptosis by pan-capase inhibitor (zVAD-fmk). Cells co-incubated for 48 h with pan-caspase inhibitor (zVAD-fmk) and tetracycline (5 × 10^−4^ M) showed a similar amount of apoptotic nuclei (cells under the bar M1) like medium control (69%). One representative experiment out of five is shown.

**Figure 3:**
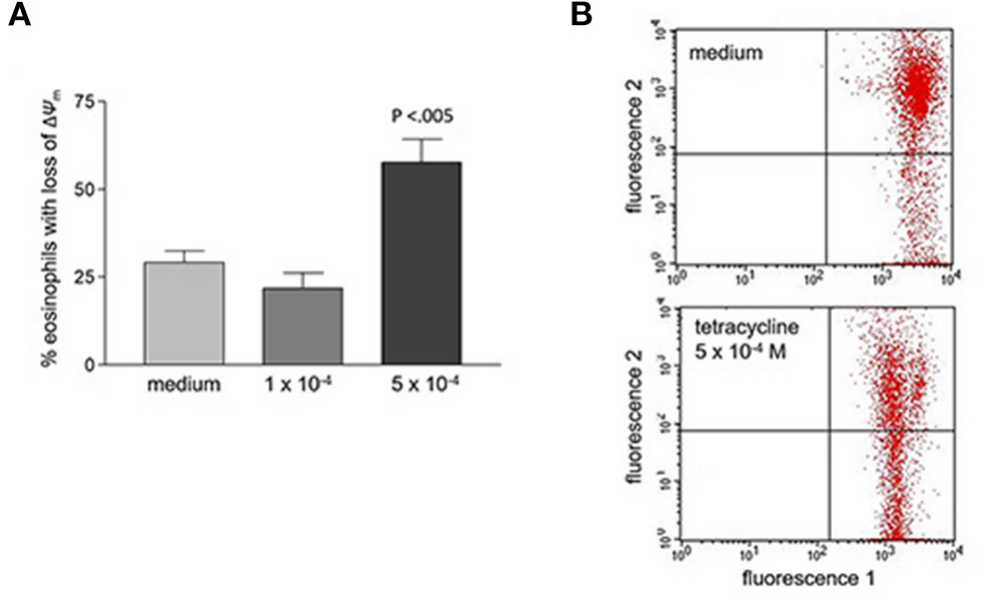
Loss of mitochondrial membrane potential (ΔΨ_m_) after 48 h stimulation with tetracycline hydrochloride. **(A)** Percentage of eosinophils with loss of ΔΨ_m_ cells is significantly increased after 48 h stimulation with 5 × 10^−4^ M tetracycline hydrochloride compared to medium (*p* < 0.005, *n* = 5). **(B)** Representative dot plot (five independent experiments) of red fluorescence 2 vs. green fluorescence 1. Compared to medium stimulation with tetracycline hydrochloride 5 × 10^−4^ resulted in a significant loss of ΔΨ_m_. Live cells with intact ΔΨ_m_ are in the upper right quadrant, apoptotic cells with decreased fluorescence 2 are in the lower right quadrant (loss of ΔΨ_m_).

Tetracycline hydrochloride, doxycycline, and minocycline significantly (*p* < 0.05 up to *p* < 0.001, *n* = 9) and dose-, and time-dependently induced eosinophil apoptosis after 24 and 48 h incubation (Figure 1). TCNs did not induce necrosis as assessed by trypan blue dye exclusion (tetracycline hydrochloride 10^−3^ M: 1% dead cells after 48 h; minocycline 10^−3^ M 14% dead cells after 48 h; doxycycline: 10% with 5 × 10^−4^ M and 2% with 10^−4^ M) except for higher concentrations of doxycycline (39% dead cells with 10^−3^ M). As tetracycline hydrochloride induced fewest necrotic eosinophils and significantly and dose-dependently induced apoptosis, we chose this TCNs for further experiments. Forty eight hours co-stimulation with the pan-caspase inhibitor zVAD-fmk abrogated tetracycline hydrochloride (5 × 10^−4^ M) induced apoptosis (Figure 2) suggesting that tetracycline hydrochloride caused caspase activation downstream of mitochondrial injury. These results implicate stress-induced signaling pathways in tetracycline-mediated apoptosis. Stimulation with tetracycline hydrochloride at 5 × 10^−4^ M resulted in a significant loss of mitochondrial membrane potential (ΔΨm; *p* = 0.005 compared to medium, *n* = 5, Figure 3A). The representative dot plot (Figure 3B) demonstrates the increase in cell numbers with decreased red fluorescence 2.

### TCNs Inhibited Prolongation of Eosinophil Survival Mediated by *staphylococcus aureus* Enterotoxins or IL-3, IL-5, and GM-CSF

**Figure 4:**
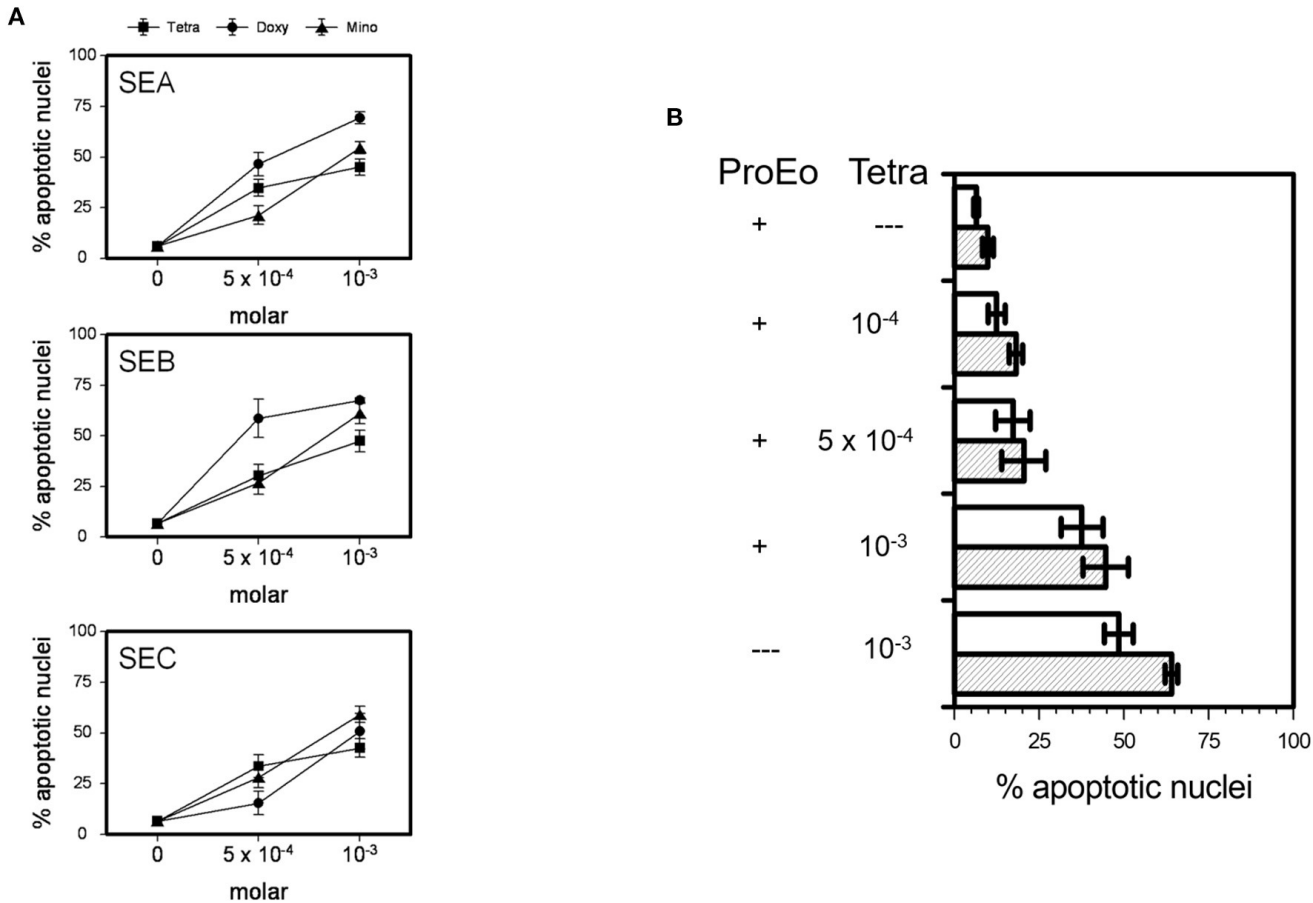
TCNs overcame the survival prolonging effects of SEA, SEB, SEC, and of key pro-survival eosinophil cytokines, i.e., a mixture of IL-3, IL-5, and GM-CSF (ProEo). **(A)** Co-incubation of 5 × 10^−4^ M or 10^−3^ M tetracycline hydrochloride (Tetra), doxycycline (Doxy), or minocycline (Mino) wit SEA, SEB, and SEC (5 μg/ml each; *p* < 0.05 up to *p* < 0.001 vs. medium control = 0). Data are presented as percentage of apoptotic nuclei ± SEM (*n* = 6). **(B)** Tetra partly abrogated the effect of ProEo. Mean percentage of apoptotic nuclei ± SEM (*n* = 5) after stimulation for 24 h (unfilled bars) and 48 h (striped bars). *P* < 0.05 up to *p* < 0.001.

All TCNs, i.e., tetracycline hydrochloride, doxycycline, minocycline, at 5 × 10^−4^ M and 10^−3^ M were able to significantly overcome the potent survival prolonging effect of SEA, SEB, and SEC (5 μg/ml each) after 24 h (Figure 4A; *p* < 0.05 up to *p* < 0.001) and after 48 h (not shown). In addition, minocycline (not shown), doxycycline (not shown), and tetracycline hydrochloride partly abrogated the effect of a pro-eosinophilic cytokine mixture consisting of IL-3, IL-5, and GM-CSF (10 ng/ml each) (Figure 4B, *p* < 0.05 up to *p* < 0.001).

### Tetracycline Hydrochloride Resulted in a Significant Production of Eosinophil Intracellular H_2_O_2_

**Figure 5:**
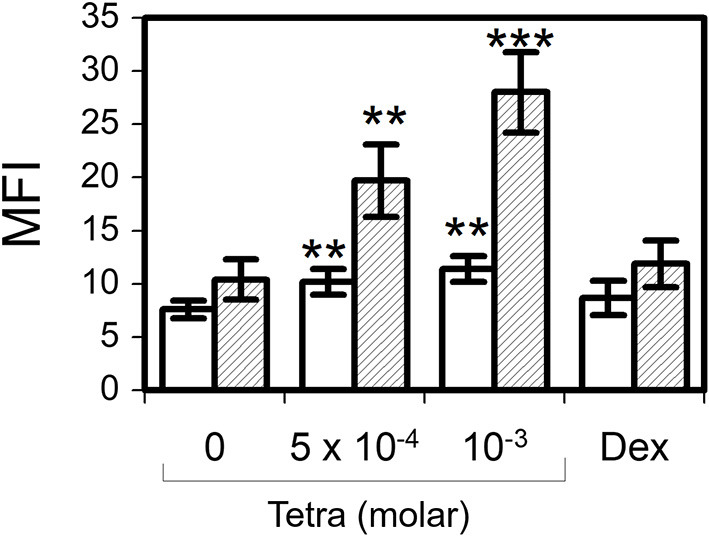
Tetracycline hydrochloride induced intracellular hydrogen peroxide production. In contrast to dexamethasone 10^−6^ M (Dex) tetracycline hydrochloride (Tetra) induced apoptosis was accompanied by a significant intracellular production of H_2_O_2_ after 2 h (unfilled bars) and 24 h (striped bars). Dihydrorhodamine assay, mean fluorescence intensity (MFI) ± SEM (*n* = 7). **p < 0.01, ***p < 0.001.

Tetracycline hydrochloride at 5 × 10^−4^ M for 2 h (*p* = 0.007) and 24 h (*p* = 0.004) resulted in a significant production of eosinophil intracellular hydrogen peroxide as well as stimulation with tetracycline hydrochloride at 10^−3^ M for 2 h (*p* = 0.003) and 24 h (*p* < 0.001) (Figure 5). In contrast, dexamethasone (10^−6^ M) was not able to induce a significant production of H_2_O_2_.

### Tetracycline Hydrochloride Induced Calcium Influx in Eosinophils

**Figure 6:**
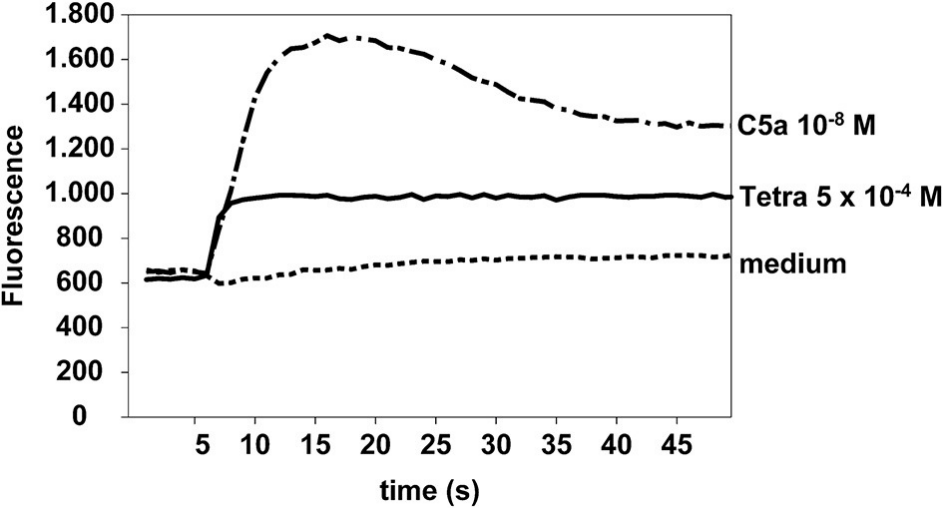
Tetracycline hydrochloride induced calcium influx. Tetracycline hydrochloride 5 × 10^−4^ M and C5a (10^−8^ M, positive control) but not medium induced a significant calcium influx in eosinophils at a very early stage. Cellular fluorescence was recorded per second for 50 s. Fluo4-AM assay.

Eosinophils were stimulated with tetracycline hydrochloride (5 × 10^−4^ M), C5a (10^−8^ M), or medium (as negative control). In contrast to medium, tetracycline and C5a (positive control) were able to increase calcium influx in eosinophils. One representative experiment out of four is shown (Figure 6).

### Modulation of Eosinophil Surface Antigens by Tetracycline Hydrochloride

**Figure 7:**
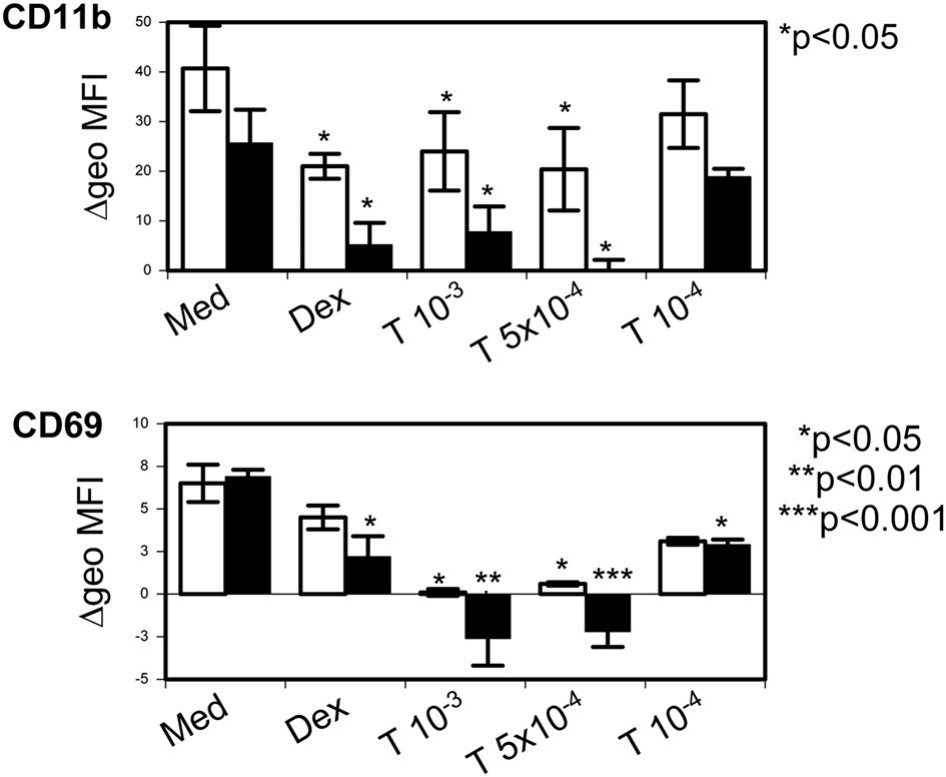
Tetracycline hydrochloride downregulated eosinophil activation markers. Tetracycline hydrochloride (T) at indicated concentrations (in molar) downregulated CD11b and CD69 after 24 h (unfilled bars) and 48 h (filled bars) stimulation. P as indicated vs. control medium = Med). Dexamethasone (10^−6^ M, Dex) was less effective. Δ geometric mean fluorescence intensity (Δgeo MFI) ± SEM (*n* = 4).

Tetracycline hydrochloride was not able to modulate eosinophil surface expression of CD63 or CD95. However, incubation for 24 h at 5 × 10^−4^ M and 10^−3^ M resulted in a significant downregulation of CD9 (*p* = 0.01 and *p* < 0.001) and of CD45 (*p* < 0.05). In contrast CD54 was upregulated but in contrast to IL-3 (10 ng/ml, *p* = 0.017) this effect did not reach statistical significance. Additionally, stimulation with dexamethasone (10^−6^ M) and tetracycline hydrochloride for 24 and 48 h was able to significantly downregulate the expression of the eosinophil activation markers CD11b and CD69 (*p* < 0.05 up to *p* < 0.001; Figure 7).

## Discussion

Unspecified non-antibiotic activities have been attributed to the beneficial effects of TCNs in several diseases. Some of these diseases are characterized by eosinophil-induced inflammation, for example asthma and bullous pemphigoid. Aim of this study was to investigate whether TCNs modulate eosinophil function and survival. Our data for the first time clearly show that TCNs, i.e., tetracycline hydrochloride, minocycline and doxycycline, significantly and dose-dependently induce eosinophil apoptosis. In contrast to necrosis, apoptosis represents a major physiologic mechanism that enables clearance of cells by phagocytosis with no or minimal damage to surrounding tissues (38). We and others have shown that delayed eosinophil apoptosis is deleterious in various allergic diseases such as atopic dermatitis, allergic rhinitis, and asthma (4, 6, 39–41). The development of drugs targeting eosinophil apoptosis is one possible strategy for the therapy of eosinophil-driven diseases.

There's some debate on the role of antibiotic therapy in the treatment of allergic disorders with concomitant bacterial infections (42, 43). Although antibiotic treatment has different outcomes in asthma, chronic rhinitis, and nasal polyposis, the impact of *S. aureus* enterotoxins in allergic diseases is increasing (44–52). *Staphylococcus aureus* enterotoxins are thought to act as superantigens, inducing production of polyclonal IgE via B-cell and T-cell activation, and triggering release of inflammatory mediators (50). Previously, we demonstrated that *S. aureus* enterotoxins are potent inhibitors of eosinophil apoptosis in atopic dermatitis (33). Therefore, we performed a series of experiments that demonstrated, that all TCNs significantly suppressed the survival-prolonging effect of SEA, SEB, and SEC. In addition, the potency of TCNs was confirmed by demonstrating, that tetracycline hydrochloride abrogated the well-known survival-enhancing effect of a mixture of pro-eosinophilic cytokines (53), namely IL 3, IL5, and GM-CSF. High concentrations of systemic and also inhaled glucocorticoids have been reported to partly reverse IL-5-afforded survival, but the effect of steroids fell off as the concentration of IL-5 increased (16, 54). It has been shown that 1 μM dexamethasone was reversed by low concentrations of GM-CSF (10 U/ml) or IL-3 (3 ng/ml) (15). In contrast, in this study tetracycline hydrochloride induced eosinophil apoptosis even in the presence of high concentrations of a mixture of IL-3, IL-5, and GM-CSF (10 ng/ml each). Tetracycline hydrochloride was chosen for further experiments as its stimulation had demonstrated fewest necrotic eosinophils compared to minocycline and doxycycline.

Caspases are activated in the interior of cells during the process to apoptosis and cleave specific death substrates. At least IL-5 is known to prolong eosinophil survival via inhibition of caspases (55). Using a pan-caspase inhibitor (zVAD-fmk) our experimental data showed that tetracycline hydrochloride-induced eosinophil apoptosis was caspase dependent.

Aside from inhibition of matrix metalloproteinases (23) some tetracycline analogues have been reported to act as inducers of apoptosis in various cancer cells (56–60). However, the precise apoptotic mechanisms are far from being understood. In this study, we showed that tetracycline hydrochloride produced an early increase in loss of mitochondrial membrane potential (ΔΨm). Similar changes of ΔΨm have been demonstrated in other cell types (61). Additionally, we found a significant upregulation of eosinophil intracellular hydrogen peroxide production 2 and 24 h after stimulation with tetracycline hydrochloride. These results clearly indicate a potent role of stress in the induction of TCNs induced eosinophil apoptosis. This is in contrast to dexamethasone induced apoptosis that is not associated with an increase of intracellular hydrogen peroxide [our data and (62)]. We and others have shown an important role of oxygen-dependent mechanisms in the regulation of eosinophil survival and apoptosis (32). Whilst tetracycline hydrochloride induced intracellular hydrogen peroxide it is tempting to speculate that TCNs may induce apoptosis by disturbing the eosinophil oxidant-antioxidant balance (32). Moreover, we demonstrated that tetracycline hydrochloride immediately increased intracellular calcium and eosinophil apoptosis has been shown to be mediated by calpains that are activated by increased intracellular calcium (63).

Addressing modulation of eosinophil surface antigen expression we found that tetracycline hydrochloride significantly downregulated activation markers such as CD11b and CD69, in addition to CD9 and CD45. We previously demonstrated that these markers are up-regulated on eosinophils by *S. aureus* enterotoxins (33). Interestingly, in that previous study the apoptosis-inducing corticosteroid dexamethasone down-regulated CD11b and CD45, but resulted in a significant increase of CD54 and CD69 (33). All surface markers that were down-regulated by tetracycline hydrochloride in this study have been implicated in the regulation of eosinophil apoptosis (64–66). These down-regulating properties of important apoptosis-regulating and activation markers on eosinophils underline the anti-inflammatory potential of TCNs.

Our data demonstrating potent anti-inflammatory effects of TCNs on eosinophils may provide a new paradigm for the choice of antibiotic treatment—if needed—in diseases associated with eosinophil inflammation. Moreover, in the future, the non-antibiotic activities of TCNs might be specifically utilized. Indeed, recent findings demonstrated that TCNs reduced airway inflammation and hyperresponsiveness in asthma animal models and that in humans, they have steroid-sparing properties in moderate persistent and “difficult to treat” asthma (51, 67–71). For the first time our results may provide an explanation for the reported non-antibiotic therapeutic effect of TCNs in bullous pemphigoid, a blistering disease with a prominent role of eosinophils that are increased in peripheral blood and in blister fluids (72, 73).

In conclusion, key findings from this study demonstrate that TCNs are very potent inducers of mitochondria-mediated and caspase-dependent apoptosis in eosinophils, down-regulate eosinophil activation markers and other surface molecules, and are even capable to overcome the effect of potent survival-enhancing cytokines such as IL-3, IL-5, and GM-CSF and *S. aureus* exotoxins. Clinically, TCNs might have less severe side effects compared to corticosteroids, which actually, are used as therapeutic option in some diseases associated with eosinophilia. Our results indicate new non-antibiotic anti-inflammatory effects of TCNs, which should be addressed in more detail in future studies.

## Data Availability Statement

The original contributions presented in the study are included in the article/supplementary material, further inquiries can be directed to the corresponding author/s.

## Ethics Statement

The studies involving human participants were reviewed and approved by Ethics Committee of Hannover Medical School. The patients/participants provided their written informed consent to participate in this study.

## Author Contributions

MG and BW conceived and planned the experiments, processed the experimental data, and performed the analysis. MG carried out the laboratory works. DW contributed to sample preparation. BW designed the figures and took the lead in writing the manuscript with support from DW and AK. All authors provided critical feedback and helped shape the research, analysis, and manuscript.

## Funding

This study was in part supported by Grant We-1912/5-2 from the German Research Foundation (DFG) to BW.

## Conflict of Interest

The authors declare that the research was conducted in the absence of any commercial or financial relationships that could be construed as a potential conflict of interest.

## Publisher's Note

All claims expressed in this article are solely those of the authors and do not necessarily represent those of their affiliated organizations, or those of the publisher, the editors and the reviewers. Any product that may be evaluated in this article, or claim that may be made by its manufacturer, is not guaranteed or endorsed by the publisher.
